# Editorial: Individual differences in addictive behaviors

**DOI:** 10.3389/fpsyg.2024.1386489

**Published:** 2024-05-22

**Authors:** Lucy J. Troup

**Affiliations:** Division of Psychology, University of the West of Scotland, Paisley, United Kingdom

**Keywords:** addiction, individual differences, technology addiction, substance use disorder, gambling addiction, mental health, comorbidity

Theories and explanations of addiction tend to focus on different and often opposing theoretical frame works rooted in specific areas of our understanding of human behavior. For example the disease model of addiction has played a significant role in the literature relating to explanations for addiction (Leshner, 1997). The focus however is shifting to a broader multidisciplinary approach. This takes into account different theoretical perspectives including for example genetics and social environmental variables which clearly have a significant role in explaining, understanding and treating addiction (Volkow et al., 2016). As well as this more inclusive approach, the scientific literature has shifted its focus toward the individual both in respect to understanding addiction and to treating addiction (Drossel et al., 2023). Further to this, as clearly expressed in this Research Topic, understanding addiction is evolving. Not only is the focus for theoretical approaches to understanding addiction shifting to the individual but it is longer the case that when we talk about addiction we are referring just to substance use disorder. As indicated in this Research Topic the role of technology in addiction especially smartphone use and interaction with the internet, has become a central theme in behavioral addiction.

## Behavioral addiction

One emerging theme for this Research Topic is the role of external factors in conjunction with internalized variables underpin compulsive and addictive behaviors relating to the use of technology. These exogenous variables intact with endogenous variables, for example socioenvironmental variables and effect the mental health of the individual in the context of addiction. The relationship between parenting style and individual differences along with biological sex differences can both be seen as variables influencing problematic gaming behavior. Rodríguez-Ruiz et al. show how parenting style contributes to scoring highly for neuroticism which is in turn linked to gaming disorder in adolescence. However in relation to excessive gaming behavior, males appear to be more susceptible than females (Rodríguez-Ruiz et al.). In another study reporting the role of parenting on cyber bullying Kim et al. note that self esteem in those reporting bullying mediated the relationship between positive parenting and smart phone addiction.

Smart phone use is also shown to be detrimental to other aspects of wellbeing for example Yang et al. show how excessive smart phone use is linked to poor sleep which in turn is a mediating variable for poor mental health. Excessive smart phone use patterns also seem to be in part at least driven by external factors. Li et al. show how visual cues and inability to exert self-control are driving habitual use patterns. In bringing together both endogenous and exogenous variables Shek et al. explored the effects of the pandemic on internet addiction. They concluded that there was a significant relationship between socioeconomic difficulty, gender and infection with COVID-19 with internet addiction (Shek et al.). In a study investigating the effects of short-form video addiction Xie et al. showed that those undergraduate students who were excessively engaging in short-form video were much more likely to result in academic procrastination. Attentional control was a driving factor in this relationship mediated by boredom (Xie et al.).

## Gambling disorder

Gambling disorder is typically explained in relation to behavioral addiction as well as a biological model of addiction (Grant et al., 2016). Interestingly Wu et al. suggest that the disease model of addiction could benefit at risk gamblers. They show that both controlled and at-risk gambling can be described in terms of illness representations, both consequential and emotional, through the lens of the commonsense model of self-regulation. Further to this at risk gambling behaviors would benefit from an educational perspective that adopts an approach that embraces gambling disorder as a illness (Wu et al.). In contrast Çitak proposes an individualistic approach to the identification and treatment of early onset gambling. Using a latent profile analysis of a Turkish population of gamblers identifying emerging profiles of gamblers from a more individualistic perspective (Çitak).

## Comorbidity of addiction with mental health

The relationship between addiction and other mental health conditions is complex. In a review of the pairwise relationships between DSM-IV diagnosis indicates that there is significant comorbidity between conditions (McGrath et al., 2020). In this Research Topic Huang et al. present data suggesting that patients with a diagnosis of late onset major mood disorder are more likely to report suicidal ideation. It is therefore important to understand comorbidity between mental health conditions and addiction to provide appropriate support.

Methodological approaches to the study of individual differences in this case biological sex are presented by Farris et al.. Suggesting that female hormonal biomarkers can be used to help understand the comorbidity between anxiety and nicotine craving. Using simple at home hormonal saliva testing alongside ecological momentary analysis they demonstrate the feasibility to collecting psychological data alongside biomarkers to help understand individual differences in smoking behaviors (Farris et al.). These data provide a methodological framework that could be applied in research in other addictive behaviors investigating comorbidity individual difference and addiction.

As behavioral addiction has garnered a great deal of interest in the literature, especially gaming, smart phone use and internet addiction, the relationship between compulsive behaviors and addiction has become a central theme, in particular how these evolve into addictive behaviors (Muela et al., 2022). In this Research Topic Zhao et al. explore how sleep disturbance and it relationship with compulsive behavior is mediated by repetitive negative thinking suggesting that targeting these thought processes within a clinical intervention may lead to a reduction in compulsive behavior in individuals.

## Substance use and addiction

Since 2018 legislation in relation to cannabis has changed significantly. It is now possible for patients in the UK who have exhausted all other treatments to be able to be prescribed medical cannabis by a specialist doctor (Torjesen, 2018). It is noted that there is very little research that addresses individual differences in cannabis use patterns and the relationship between medical and recreational cannabis users (Pacula et al., 2016). Ciesluk et al. address this lack of research in individual differences in cannabis use behaviors in the UK, considering its recent legislative changes. Whilst they note significant differences in recreational cannabis users when compared to medical cannabis users, with trait anxiety scores being similar between the two groups, recreational cannabis users reported high levels of state anxiety. There were also differences in age, medical cannabis users being older and differing in their motives for using. However they also show overlap in some motives for using (Ciesluk et al.).

Finally, an interesting study that looks at a consequential outcome of prolonged opium smoking in an Iranian population. Massah et al. present some interesting data that indicates that opium smoking can lead to physical changes in individuals over prolonged time leads to impairment in range of motion and muscle strength in the neck.

This Research Topic reflects the evolving pattern of addiction and its consequences and highlights some important areas that clearly would benefit from more research. It is essential that we consider individual difference as a key part of both theory and treatment in future research in addiction.

## Author contributions

LT: Conceptualization, Data curation, Formal analysis, Funding acquisition, Investigation, Methodology, Project administration, Resources, Software, Supervision, Validation, Visualization, Writing—original draft, Writing—review & editing.

## References

[B1] Drossel G. Brucar L. R. Rawls E. Hendrickson T. J. Zilverstand A. (2023). Subtypes in addiction and their neurobehavioral profiles across three functional domains. Transl. Psychiatry 13:1. 10.1038/s41398-023-02426-1 37072391 PMC10113211

[B2] Grant J. E. Odlaug B. L. Chamberlain S. R. (2016). Neural and psychological underpinnings of gambling disorder: a review. Prog. Neuro-Psychopharmacol. Biol. Psychiatry 65, 188–193. 10.1016/j.pnpbp.2015.10.007 26497079

[B3] Leshner A. I. (1997). Addiction is a brain disease, and it matters. Science 278, 45–47. 10.1126/science.278.5335.45 9311924

[B4] McGrath J. J. Lim C. C. W. Plana-Ripoll O. Holtz Y. Agerbo E. Momen N. C.. (2020). Comorbidity within mental disorders: a comprehensive analysis based on 145 990 survey respondents from 27 countries. Epidemiol. Psychiatr. Sci. 29:e153. 10.1017/S2045796020000633 32782057 PMC7443806

[B5] Muela I. Navas J. F. Ventura-Lucena J. M. Perales J. C. (2022). How to pin a compulsive behavior down: a systematic review and conceptual synthesis of compulsivity-sensitive items in measures of behavioral addiction. Addict. Behav. 134:107410. 10.1016/j.addbeh.2022.107410 35780595

[B6] Pacula R. L. Jacobson M. Maksabedian E. J. (2016). In the weeds: a baseline view of cannabis use among legalizing states and their neighbours. Addiction 111, 973–980. 10.1111/add.13282 26687431 PMC5216038

[B7] Torjesen I. (2018). Medical cannabis will be available on prescription in UK from autumn. BMJ 362:k3290. 10.1136/bmj.k3290 30049690

[B8] Volkow N. D. Koob G. F. McLellan A. T. (2016). Neurobiologic advances from the brain disease model of addiction. N. Engl. J. Med. 374, 363–371. 10.1056/NEJMra1511480 26816013 PMC6135257

